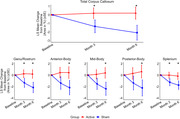# OVERTURE Phase 2 MRI Analysis Demonstrates Reduced Corpus Callosum Atrophy in Alzheimer's Disease

**DOI:** 10.1002/alz.085446

**Published:** 2025-01-09

**Authors:** Xiao Da, Evan Hempel, Mihaly Hajos, Ralph Kern, Aylin Cimenser

**Affiliations:** ^1^ Cognito Therapeutics, Cambridge, MA USA; ^2^ Department of Comparative Medicine, Yale University School of Medicine, New Haven, CT USA

## Abstract

**Background:**

Recent research by Da et al. (2023) has demonstrated that non‐invasive gamma sensory stimulation can reduce brain white matter atrophy and myelin content loss. The impact on the Corpus Callosum (CC), the brain's largest commissural white matter tract essential for hemispheric connectivity, remains unexplored. Diffusion Tensor Imaging (DTI) studies (Delvenne et al. 2023), have identified variations and alterations in the CC's tract bundles in Alzheimer's disease (AD). We conducted an analysis of CC changes in patients along the Alzheimer’s continuum targeting both the overall and regional structural alterations.

**Method:**

In this study, we used neuroimaging data from the OVERTURE clinical trial (NCT03556280). Participants were randomized 2:1 (Active: Sham) to receive daily, 1‐hour, audio and visual stimulation at gamma frequency or sham stimulation for 6 months. Magnetic Resonance Imaging (MRI) assessments were performed at baseline, month 3, and month 6. T1w MRI images were processed using the Yuki module within the Automated Registration Toolbox (ART) (Ardekani et al., 2014) with CC segmentation in the midsagittal plane (MSP) and its five subregions (Hampel et al., 1998). The analysis was conducted on participants with valid data for the total MSP CC and its subregions (N=50). Using Bayesian linear mixed‐effects modeling, we examined the changes in the area of the total MSP CC and its subregions.

**Result:**

The active group demonstrated preservation of the total CC area and its subregions after six months of treatment. Differences in percentage changes between active and sham groups were: Total CC area (2.28±0.87%, p<.02), genu/rostrum (2.36±0.90%, p<.02), anterior‐body (2.64±1.26%, p<.04), mid‐body (2.79±1.18%, p<.03), posterior‐body (2.87±1.41%, p<.05) and splenium (1.58±0.73%, p<.04). Significant differences between the two groups were also observed in total CC area, genu/rostrum, and splenium at month 3.

**Conclusion:**

OVERTURE Phase 2 MRI outcomes demonstrate that six months of non‐invasive gamma sensory stimulation may reduce the progression of CC atrophy in individuals with mild‐moderate AD, potentially preserving the integrity of interhemispheric communication. We are currently conducting a phase 3 clinical trial titled HOPE (NCT05637801) to evaluate the efficacy and safety of Cognito’s medical device.